# Olive (*Olea europaea* L.) Genetic Transformation: Current Status and Future Prospects

**DOI:** 10.3390/genes12030386

**Published:** 2021-03-09

**Authors:** Elena Palomo-Ríos, Isabel Narváez, Fernando Pliego-Alfaro, José A. Mercado

**Affiliations:** Instituto de Hortofruticultura Subtropical y Mediterránea “La Mayora”, Departamento de Botánica y Fisiología Vegetal, Universidad de Málaga, 29071 Málaga, Spain; narvaez@uma.es (I.N.); ferpliego@uma.es (F.P.-A.); mercado@uma.es (J.A.M.)

**Keywords:** *Agrobacterium rhizogenes*, *Agrobacterium tumefaciens*, biolistic, olive, somatic embryogenesis, transgenic plant

## Abstract

Olive (*Olea europaea* L.) is the most characteristic and important oil crop of the Mediterranean region. Traditional olive cultivation is based on few tens cultivars of ancient origin. To improve this crop, novel selections with higher tolerance to biotic and abiotic stress, adaptable to high-density planting systems and resilient to climate change are needed; however, breeding programs are hindered by the long juvenile period of this species and few improved genotypes have been released so far. Genetic transformation could be of great value, in the near future, to develop new varieties or rootstocks in a shorter time; in addition, it has currently become an essential tool for functional genomic studies. The recalcitrance of olive tissues to their in vitro manipulation has been the main bottleneck in the development of genetic transformation procedures in this species; however, some important traits such as fungal resistance, flowering or lipid composition have successfully been manipulated through the genetic transformation of somatic embryos of juvenile or adult origin, providing a proof of the potential role that this technology could have in olive improvement. However, the optimization of these protocols for explants of adult origin is a prerequisite to obtain useful materials for the olive industry. In this review, initially, factors affecting plant regeneration via somatic embryogenesis are discussed. Subsequently, the different transformation approaches explored in olive are reviewed. Finally, transgenic experiments with genes of interest undertaken to manipulate selected traits are discussed.

## 1. Introduction

Olive (*O. europaea* L.) is one of the most important fruit crops, and the main oil crop, in the Mediterranean basin, although its cultivation has extended throughout the world. *O. europaea* ssp. *europaea* belongs to the family *Oleaceae* and includes cultivated (var. *europaea*) and wild olives (var. *sylvestris*) [1]. The species is diploid (2n = 2X = 46), although many authors have described polyploidy in both mutants [2] and natural populations [3,4]; its genome size is ca. 1800 Mb in both cultivated and wild varieties [5]. 

Olive is a long-lived tree with a long juvenile stage that can reach up to ten years. Although there have been several breeding programs devoted to obtaining new varieties with desired characters, the long juvenile period of this species has hampered this objective, and most traditional olive cultivars had been the result of ancient grower selections [6]. Some management techniques can be used to reduce the juvenile period with different success rates depending on the genotype; however, these practices are not fast enough to meet the industry requirements. Consequently, in the last 20 years, only relatively few numbers of varieties have been released as a result of conventional breeding [7]. The first crossbreeding programs in olive were developed between 1960 and 1971 in Israel [8] and Italy [9]; since then, more than 50 countries maintain around 100 regional, national and international olive collections [10,11]. The main aims in olive breeding are early bearing, high yield, and adaptability to the high-density planting system for mechanical harvesting. More recently, programs also include resistance to Verticillium wilt and other diseases among their targets [12].

Due to the constraints imposed by the reproductive behavior and high level of heterozygosity of woody species, genetic transformation has emerged as a powerful tool for the genetic improvement of fruit trees, allowing the introduction of selected traits into elite cultivars in a short period of time [13]. Efficient procedures for in vitro regeneration are needed to develop successful genetic transformation protocols in any species. In this regard, olive is a recalcitrant species to in vitro manipulation [14]; however, in the last two decades, a small number of studies reported the development of olive genetic transformation protocols, which have been used for obtaining transgenic plants expressing reporter genes and even transgenes of interest, although in most cases transformed explants were of juvenile origin. The importance of this technology will increase in the near future with the advancement in the olive genome sequence projects that are currently underway [15,16,17], which could allow the identification of genes of interest for future breeding programs. In addition, protocols for the regeneration of somatic embryos (SE)from explants of adult origin are needed to develop new cultivars or rootstocks useful for breeders.

In this review, we describe the main advances in the genetic transformation of this species, the current and future challenges of this technology and its potential use for olive improvement. Initially, in vitro tissue culture protocols for olive regeneration via somatic embryogenesis are discussed since most transformation studies use somatic embryos as initial explants.

## 2. Somatic Embryogenesis

As in many other woody species, somatic embryogenesis, a process in which embryos derive from somatic cells, is the most used system for the in vitro regeneration of olive plants. The genotype and ontogenetic age of the material are determinant factors in the success of the process. Different explants of juvenile (seed derived) origin have been employed for this purpose, such as immature zygotic embryos [18], radicles from mature embryos [19,20], cotyledon fragments [21,22], and seedling roots [23,24]. In the first study [18], full immature embryos, 75 days after pollination (dap.), from cvs. “Morailo”, “Frantoio” and “Dolce Agogia” were used to induce somatic embryogenesis. The embryogenic potential of these explants could be maintained by storing the fruits at 14–15 °C for 2–3 months [25]. Cotyledons from 60 to 90 dap. immature embryos, depending on the genotype, were also suitable for the establishment of embryogenic cultures [21]. Similarly, cotyledons from 70 dap. embryos from two Tunisian cultivars were successfully used to induce somatic embryogenesis [26]. In the case of mature zygotic embryos, radicle segments showed a high embryogenic response, while cotyledonary segments yielded poor results, both in wild [19] and cultivated olive [20]. Although other protocols have also been successful, the use of radicle segments from mature zygotic embryos appears to be the preferred choice to obtain successful results [27,28,29,30,31].

The culture media most widely used for embryogenic induction are OM (olive medium) [32] and its modifications, i.e., OMc [19,20,31,33,34], and olive cyclic embryogenesis (ECO) medium (a modified OMc with a lower ionic strength) [35]. Murashige and Skoog (MS) medium [36] and 1/2 MS have also given good results [18,25,26,37]. Contrary to these studies, a different salt formulation, Schenk and Hildebrandt (SH) [38], has also been employed, but only cv. “Frangivento” could be maintained in culture [21]. The results obtained in these reports varied greatly with the genotype used, which could reflect different nutritional requirements.

**Figure 1:**
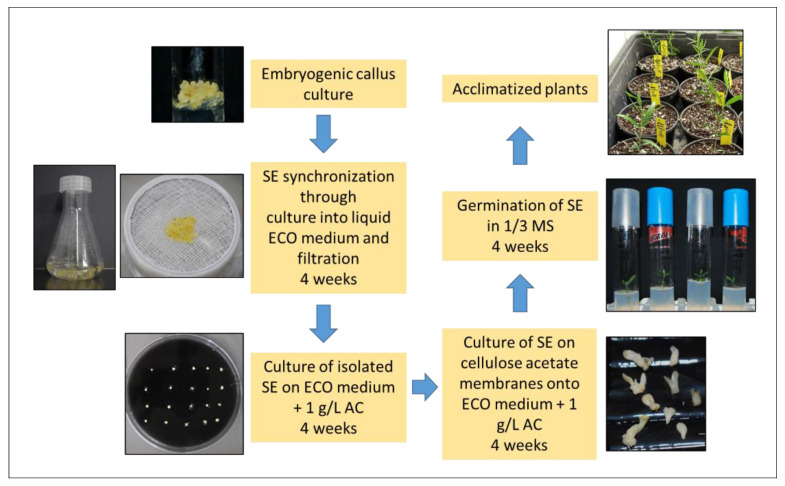
Images of the steps of olive somatic embryos (SE) maturation and plant regeneration protocol [28].

Regarding the hormonal balance, most studies found that the addition of auxins and cytokinins to the culture medium was essential in the induction phase, keeping a high auxin/cytokinin ratio [19,20,21,22,23,24,29,34,37], followed by a reduction in auxin concentration [19,20] or even auxin elimination [21,34] for somatic embryo expression and maintenance. Generally, explants are incubated in darkness for embryogenic induction. The maturation and conversion of SE are the main bottleneck in olive due to the low number of SE converted into plants [39,40]. Few reports have addressed SE maturation in olive. Sucrose has been found to be better than glucose for maturation [34]. Other treatments such as chilling and growth regulator inhibitors have not given good results [41]. Abscisic acid has been used to synchronize embryo maturation [41]. In a different maturation approach, embryogenic masses were cultured into liquid medium followed by filtering through a 3 × 3 mm mesh, and the subsequent structures of lower size were cultured on maturation medium (ECO medium without plant growth regulators (PGRs), supplemented with 1 g/L activated charcoal (AC) [28]. Maturated embryos of higher quality were obtained when globular embryos were incubated for 4 weeks onto this medium, followed by 4 weeks over cellulose acetate semi-permeable membranes [28]. This treatment could decrease embryo water potential and increase solute accumulation, improving the germination percentage. Figure 1 shows the steps followed in this protocol, from SE to obtaining acclimatized plants.

MS mineral formulation [24,37] and modified MS of lower strength, 1/3 or 1/4 MS [23,25,28], have been used to germinate mature olive embryos. A low concentration of cytokinin in the culture medium is also recommended by some authors during this process [18,23,34].

Somatic embryogenesis from mature (after plant has reached reproductive capacity) tissues of elite cultivars is one of the main challenges of olive tissue culture. Some successful studies have been reported, although the methodology and efficiency strongly depend on the genotype used. Somatic embryogenesis from cultivars “Canino” and “Moraiolo” has been obtained using a double regeneration system [42]. In the first stage, shoot organogenesis was induced from petioles of micropropagated plants. The cells from these shoots suffered a rejuvenation process that allowed the formation of embryogenic cultures in the second phase, when the small new leaves formed from the adventitious buds were used as explants. Leaves and petioles from plants rejuvenated through in vitro culture have also been used to obtain embryogenic callus from cvs. “Dahbia” [43] and “Picual” [44]. The in vitro rejuvenation of adult tissue is not strictly necessary to obtain embryogenic callus. Somatic embryogenesis from leaves and petioles obtained from adult plants of wild olive growing in the greenhouse has also been observed [45]. In the case of wild olive cvs. “StopVert”, “OutVert”, “Ac-18” (resistant to *Verticillium dahliae*) and “Ac-15” (susceptible to this fungus), shoot apex (apical meristem with one or two leaf primordia), petiole and leaf sections from in vitro micropropagated shoots were used as explants, although only the shoot apex showed a positive response in two (“StopVert”, “Ac-18”) out of the four genotypes tested [46]. The strong genotype effect on SE induction in explants of adult origin has previously been reported in olive [47] and other woody perennials [48]. Different culture media have been used for embryogenic induction in adult explants, e.g., OM formulation [42,44], MS and 1/2 MS [43,45,46]. Contrary to observations in explants of juvenile origin, a low auxin-cytokinin ratio was required in most cases to induce embryogenesis from adult material, ca. in the case of cvs. “Canino” and “Moraiolo”, the medium was supplemented with N^6^-(2-Isopentenyl)adenine (2iP) (0.5 µM), N^6^-benzyladenine (BA) (0.44 µM) and indole-3-butyric acid (IBA) (0.25 µM) [42]; a high concentration of thidiazuron (TDZ) (30 µM) in combination with naphthalene acetic acid (NAA) (0.5 µM) has been employed in other genotypes [43,44,46]. By contrast, embryogenic cultures in wild olive explants have also been obtained in the presence of IBA (12.25 µM) and zeatin (4.56 µM) [45]. Despite these successful reports, the percentages of somatic embryogenesis induction obtained are, in general, lower than those obtained with juvenile explants. Proembryogenic masses (PEM) from explants of adult origin can be maintained in the same medium used for culture initiation [42,46], in OM or ECO media supplemented with 2iP (0.5 µM), BA (0.44 µM) and IBA (0.25 µM); for embryo maturation, PEM of cvs. “Canino” and “Moraiolo” were placed on filter paper soaked with liquid basal OMc medium with subsequent transfer to the same medium in semisolid form and supplemented with AC (1 g/L); under these conditions, several cycles of secondary embryogenesis could be obtained by monthly subcultures. Embryo germination required culture in liquid OMc medium, under agitation at 80 rpm, and supplemented with zeatin (1.3 µM). In the case of wild olive genotype “StopVert”, maturation and germination conditions were as previously described for juvenile material and explained in Figure 1 [28,46].

## 3. Genetic Transformation Procedures

Biolistic and *Agrobacterium tumefaciens* mediated transformation have been reported in olive, using SE as explants in both cases. *A. rhizogenes* has also been used to obtain olive chimeric plants with transgenic roots.

### 3.1. Biolistics

Early works in olive genetic engineering aimed to develop a transient transformation protocol using a biolistic approach. Large-size somatic embryos (>5 mm length), cv. “Canino”, were transformed with two plasmids, pZ085 and pCGUδ0, both containing the β-glucuronidase (*GUS*) reporter gene [49]. The use of different particles, tungsten vs. gold, and different bombardment devices, Particle Inflow Gun (PIG) vs. PDS-1000/He, did not affect the results; a GUS histological signal was obtained with each experimental condition when a 580-kPa shoot pressure was applied. Later, the optimal conditions for the transient transformation of SE derived from a mature “Picual” zygotic embryo using the PDS-1000/He system were analyzed [35]. The best results were obtained when a target distance of 6 cm and a bombardment pressure of 900-psi were employed, using gold particles of about 1 µm. The plasmid pCGUΔ1, containing the GUS gene under the control of the sunflower ubiquitin promoter, yielded higher transient transformation rates than pGUSInt containing the CaMV35S-GUS chimeric gene. Similar results had been previously obtained [49] when SE of a small size were used for bombardment. Although transgene expression was detected in the embryogenic callus 12 weeks after bombardment, a uniform selection of the transgenic material was not possible, and transgenic plants were not regenerated [35].

### 3.2. A. Rhizogenes Transformation

*A. rhizogenes* mediated transformation can be used to produce composite plants formed by transgenic roots attached to a wild-type shoot, providing an excellent tool for studying root biology [50]. The first application of this technology in olive was reported using in vitro grown shoots of “Dolce Agogia” and “Moraiolo” [51]. The basal surface of the shoots was wounded and infected with a razor blade previously submerged in an *A. rhizogenes* culture; then, the shoots were cultured in ½ MS medium. Roots appeared 10 days after infection. Later, the protocol was modified, supplementing the bacterial culture with 1 mM putrescine to increase rooting [52]. Using this protocol, more than 50% of the inoculated shoots produced roots, but transformation efficiency was lower since only a few plants (<20%) had agropine-positive roots.

In a different approach, olive plants from cv. “Manzanillo” growing in the glasshouse were used for *A. rhizogenes* transformation [53]. The roots of these plants were trimmed to 4–5 cm and infected with the wild *A. rhizogenes* super rooting strain 232. Inoculated plants showed enhanced growth and superior reproductive behavior than non-infected plants. The authors suggested that this technique could be used as a standard agricultural practice, although further studies on this issue have not been reported.

### 3.3. A. Tumefaciens Transformation

The efficiency of the *A. tumefaciens* transformation system depends on multiple factors, such as bacterial strain, type of explant, selectable marker gene and approach for the selection of transgenic cells and the regeneration system [54]. Along this line, the choice of the selection agent and its concentration during the selection phase is particularly relevant, e.g., a low amount of antibiotic or herbicide for selection could lead to the appearance of chimeric tissues containing transgenic and non-transgenic cells, or escapes; on the contrary, an excessively high concentration could provoke the death of transgenic cells and prevent their regeneration, especially during the early selection phase. The antibiotic kanamycin has been recommended to select olive cells transformed with the *neomycin phosphotransferase* (*nptII*) gene in the range 50–100 mg/L; it is necessary to previously evaluate the antibiotic concentration and the time of culture of explants to obtain the best results because each explant has a different sensitivity to kanamycin [54]. In this regard, the use of different aminoglycoside antibiotics, i.e., paromomycin, kanamycin and neomycin, has been studied for the selection of an olive embryogenic culture obtained from a radicle of a seed of the cv. “Picual” [35]. Embryogenic masses and isolated embryos showed a high tolerance to paromomycin and kanamycin when cultured on solid medium, being the optimal concentration required to impair callus growth higher than 200 mg/L. However, a very low concentration of paromomycin, 3 mg/L, was sufficient to restrain the growth of callus cultured in liquid medium [35]. The herbicide phosphinotricin (PPT) is commonly used as a selectable agent in transformation experiments with the *bar* or *pat* marker genes from *Streptomyces spp*. The growth of olive embryogenic callus in solid medium was partially inhibited at 2.5–10 mg/L PPT [55]. These authors recommended selection of three weeks in liquid medium supplemented with 10 mg/L PPT as the most effective treatment.

The first studies on the stable transformation of olive via *A. tumefaciens* were reported using SE from adult origin, cv. “Canino” [54]. These embryos were inoculated with *A. tumefaciens*, strain LBA4404, carrying the binary plasmid pBIN19 that contains the selectable gene *nptII* [54]. Embryogenic masses were inoculated with the bacterial suspension and incubated for 48 h in flasks under agitation, 80 rpm, in darkness. Afterwards, embryogenic masses were rinsed in sterile distilled water, blotted dry over sterile paper, and cultured on solid embryogenic medium [42] containing 250 mg/L cefotaxime for a month. The authors pointed out that cefotaxime was the best antibiotic for controlling *A. tumefaciens* growth, and its use improved plant regeneration [54]. Afterwards, the embryogenic masses were cultured on the same medium, but supplemented with the selectable agent kanamycin at 100 mg/L. To control bacterial growth and improve the selection process, drops of the melted medium were added over the embryogenic masses. Later, these masses and isolated embryos were cultured in liquid medium supplemented with 0.3 mg/L zeatin, under light. Green embryos obtained after 15–20 days were cultured individually on solid medium containing 150 mg/L kanamycin, in darkness, to produce new SE. Afterwards, SE derived from the green embryos that had survived the selection phase were cultured in liquid medium supplemented with zeatin, without selectable antibiotics, under light to recover plants. Unfortunately, the transformation rate using this protocol was not reported.

**Figure 2:**
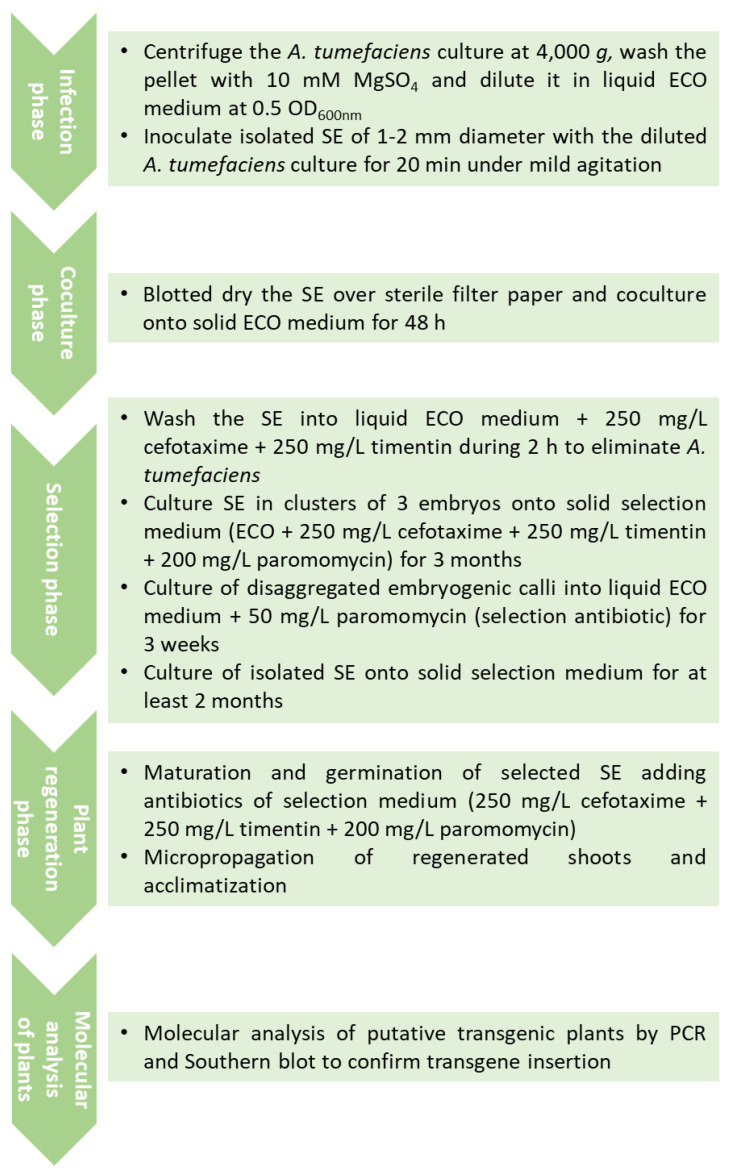
Workflow chart of *A. tumefaciens*-mediated transformation of olive SE using the protocol described by [56].

In a different approach, isolated globular SE were employed for inoculation with *A. tumefaciens* and a selection phase in liquid medium was introduced [56]. Briefly, SE (1–2 mm diameter) were incubated with the diluted bacterial suspension, blotted dry over sterile filter paper, and cultured on solid embryogenic medium (ECO) for 48 h in darkness. After co-culture, SE were washed for 2 h with liquid ECO medium containing cefotaxime and timentin, each at 250 mg/L, to restrict bacterial growth. Then, the globular embryos were transferred to solid selection medium, ECO medium with cefotaxime, timentin and paromomycin at 200 mg/L. After 3 months of culture, disaggregated embryogenic calli resistant to paromomycin were cultured in liquid ECO medium supplemented with 50 mg/L paromomycin for 3 weeks. Then, calli were filtered and globular embryos were cultured individually on solid ECO selection medium with 200 mg/L paromomycin for at least two additional months. Somatic embryos surviving this exposure were considered to be transgenic. In this study, the authors evaluated the effect of bacterial strain and binary vector on transformation efficiency. The best results were obtained with the hypervirulent *A. tumefaciens* strain AGL1 and the plasmid pBINUbiGUSInt, a combination that yielded transformation rates higher than 10% based on GUS assays. Additionally, the incubation of the *Agrobacterium* inoculated embryos in small clusters of three units during the selection in solid medium slightly increased the transformation efficiency. This treatment probably allowed transformed embryos with a low level of expression of the selectable marker gene to withstand the selection pressure. Maturation was carried out using cellulose acetate membranes [28], while for germination, a basal medium with low sucrose and a diluted salt formulation [28,57], as previously described for non-transgenic material, was used, although it was supplemented with the same concentration of selective antibiotic to assure the recovery of transgenic plants. The transgenic nature of olive plants was confirmed by both polymerase chain reaction (PCR) amplification and Southern blot analysis. All putative transgenic plants showed PCR amplification of a fragment of the *nptII* gene; regarding the Southern blot analysis, 1–3 copies of the transgene were found in the different transgenic lines analyzed [56]. One of the embryogenic lines used for transformation did not yield any transgenic callus [56], which points out the important effect of the genotype in the transformation process. A workflow chart of this protocol is shown in Figure 2.

The size of the transgenes inserted could affect regeneration efficiency; hence, the protocol described above was adapted to different constructs by employing a progressive selection strategy, which would be less stressful for the globular embryos than the continuous high selection pressure used in the original protocol [58]. It is then recommended to start the selection phase using a medium supplemented with 50 mg/L paromomycin and progressively increase the concentration to reach 150 mg/L at the end of the process.

**Figure 3:**
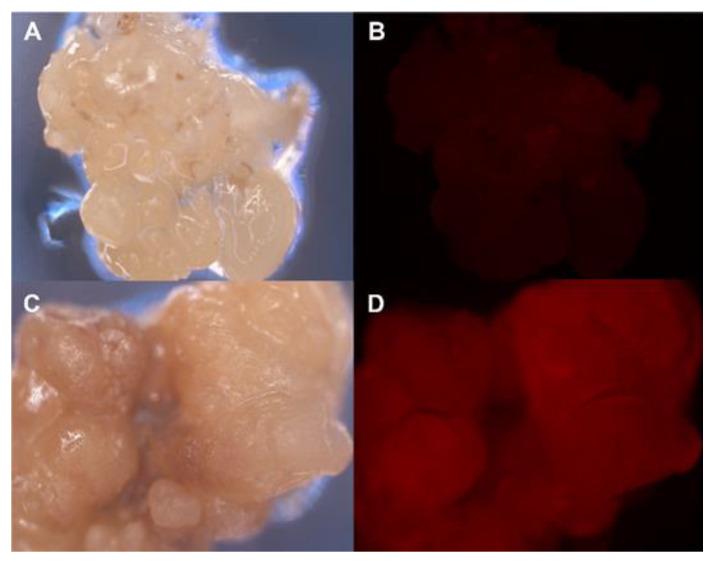
Red fluorescence in olive embryogenic callus transformed with the *DsRed* marker gene. (**A**,**B**) Control non-transformed callus. (**C**,**D**) *DsRed* transformed embryogenic callus. Pictures were taken under white light (**A**,**C**) and fluorescent light using a DsRed excitation filter (**B**,**D**).

Marker genes encoding fluorescent proteins have been proposed as an alternative to avoid the use of selectable antibiotics or to help in the early selection of transgenic cells [59,60]. In embryogenic cultures, these genes could allow the use of lower selection pressures and shorter selection times, yielding higher transformation efficiencies [61,62]. The utility of green (*gfp*) and red (*DsRed*) fluorescent marker genes in the *A. tumefaciens* transformation of olive SE has been analyzed [63]. Both proteins could be detected in transformed embryos after *Agrobacterium* infection using an epifluorescence microscope. Embryos transformed with the *DsRed* gene showed the highest fluorescent signal throughout the transformation procedure (Figure 3); moreover, a signal could also be detected in the leaves and roots of regenerated plants using a confocal microscope. The DsRed fluorescent signal overcame the threshold level to be detected 6 weeks after *Agrobacterium* inoculation of the embryos and maintained a stable signal thereafter. The combined use of DsRed and antibiotic selection is a promising approach to improve olive transformation rates.

## 4. Genetic Transformation with Genes of Interest

The genetic transformation technologies developed in olive have been applied to improve different traits that are difficult to accomplish with traditional breeding. The main objective in most research works has been to increase tolerance to fungal pathogens, particularly Verticillium wilt and *Spilocea oleagina*, but other important traits have also been modified (Table 1).

### 4.1. Fungal Tolerance

Transgenic olive plants with higher tolerance to *Spilocaea oleagina*, the causal agent of peacock leaf spot, by overexpressing an *osmotin* gene from tobacco, have been obtained [64]. Embryogenic cultures were inoculated with *A. tumefaciens* strain LBA4404 containing the *osmotin* gene under the control of the constitutive promoter *CaMV35S* [54]. This gene encodes for one of the Pathogen Related Proteins family 5 (PR5), present in all plant genomes analyzed so far. Transgenic plants were evaluated for 10 years in field trials, showing a phenotype similar to control non-transformed plants, although the leaf lamina was slightly narrower. High amounts of osmotin were found in the epidermal and subepidermal cells of transgenic plants [70]. These plants showed higher resistance to *S. oleagina* than controls, but unexpectedly they were more susceptible to other pests, such as the insects *Otiorrynchus cribricollis* G. and *Lychtensia viburnii* S. [71].

Fungal resistance has been achieved in other species through the ectopic expression of genes from fungal or bacterial origin encoding antimicrobial proteins, such as chitinase or glucanase from *Trichoderma harzianum* [72,73] or the antifungal protein AFP from *Aspergillus giganteus* [74]. AFP is a small protein that disturbs the integrity of the plasma membrane and inhibits chitin biosynthesis in sensitive fungi [74]. This protein has been expressed in different crops, improving their resistance to fungal diseases, e.g., rice [75,76], wheat [77,78] and pearl millet [79]. In olive, globular SE derived from a seed of “Picual” were transformed with the AGL1 *A. tumefaciens* strain harboring the *afp* gene under the control of the *CaMV35S* promoter [65]. The transgenic plants obtained did not show any phenotypic difference with the control when growing in the greenhouse. Plants from five independent transgenic lines were tested against *Verticillium dahliae*, defoliant pathotype, and the necrotrophic pathogen *Rosellinia necatrix*, the causal agent of white root rot in fruit trees. Constitutive *afp* expression did not affect the response of olive plants to Verticillium, and transgenic plants were as susceptible to the pathogen as the control. However, two transgenic lines showing the highest levels of *afp* expression displayed an enhanced degree of incomplete resistance to *R. necatrix*. These results indicate that AFP may operate in a species-specific manner, as previously suggested [80], being effective against some ascomycetes at low concentration but not against others.

The *NPR1* gene, a key regulator of the systemic acquired resistance (SAR), a resistance response at the whole plant level occurring after a previous localized exposure to a pathogen, has been used to enhance resistance to a broad spectrum of pathogens [81]. Several species were transformed with the *AtNPR1* gene from *Arabidopsis thaliana* to achieve this goal, e.g., tomato plants with enhanced resistance to Fusarium wilt and bacterial wilt [82], cotton plants with improved resistant to several fungi (Fusarium, Rhizoctonia and Alternaria) and the reniform nematode [83]. Transgenic *AtNPR1* cotton plants were also resistant to non-defoliating (ND) strains of *V. dahliae*, but not to defoliating (D) ones [84]. In olive, three transgenic lines expressing the *AtNPR1* gene under the control of the *CaMV35S* promoter were recovered and tested against *V. dahliae*, D and ND pathotypes, and *R. necatrix* [58]. Two of these lines showed an overexpression of a PR1-homologue gene, but endochitinase activity levels were not affected. The infection assay with *V. dahliae* D pathotype did not yield any difference between transgenic and control, non-transgenic, plants, and all *Verticillium* inoculated plants eventually died. However, the transgenic line with the highest *AtNPR1* expression level showed less severe symptoms than control plants after inoculation with ND pathotype. Regarding the infection test with *R. necatrix*, all the transgenic lines showed a slower disease progression than control plants, but it was not sufficient to control the disease.

### 4.2. Resistance to Abiotic Stress

Besides its antifungal activity, the transgenic expression of osmotin genes turned out to induce a better response to different abiotic stresses. Transgenic olive plants expressing the tobacco osmotin gene [54] displayed better performance when subjected to basal irrigation conditions in the field compared to control plants, which eventually died with this irrigation regime [71]. Under in vitro conditions, osmotin transformed shoots cultured in medium supplemented with PEG 8000 (1, 2 and 4%) for 28 days showed normal growth, accompanied by the accumulation of proline and enzymes related to drought stress tolerance in leaves, while non-transformed plants presented symptoms of damage and reduced growth [66]. The results obtained following the analysis of these plants confirmed that osmotin protects the plant membrane from lipid peroxidation, conferring increased tolerance to drought stress. Additionally, osmotin also induced cold protection in these olive transgenic plants by altering programmed cell death and cytoskeleton organization [67].

### 4.3. Olive Oil Quality

Genetic transformation has been used to study olive oil quality, particularly the role of the 13-hydroperoxide lyase gene (*13-HPL*) in volatile formation [68]. It is believed that this enzyme plays a key role in the lipoxygenase pathway, regulating the C6 volatile content mainly responsible for virgin olive oil aroma [85]. Globular SE were transformed with binary vectors for the constitutive overexpression and interfering RNA (RNAi) silencing of the *13-HPL* gene [68]. The 13-HPL enzyme activity increased in leaves of overexpressed transgenic lines, as well as the C6 volatile compounds, whereas opposite results were found in leaves of silenced plants, showing a significant reduction in 13-HPL activity and C6 volatile content, linked to an increase in the amount of C5 compounds. Although the profile compounds of olive oil from transgenic plants have not been reported, it is likely that results similar to those described for leaves could be obtained, since previous studies showed that leaves, fruit mesocarp and calli derived from cotyledons displayed a similar lipid composition [86]. Interestingly, 13-HPL silencing severely reduced plant growth and vigor (Elena Palomo-Ríos, personal communication, University of Málaga, August 2020), as observed in some *hpl* rice mutants [87,88].

### 4.4. Plant Architecture/Flowering

Modern agricultural practices in olive include high planting density for mechanical pruning and harvesting; plants of reduced size and altered architecture are required for these new orchards. To meet these requirements, both immature zygotic embryos of the cv. “Moraiolo” and embryogenic cultures from mature tissue of cv. “Canino” were transformed with *rolABC* genes from *A. rhizogenes* [64] using the LBA4404 *A. tumefaciens* strain. Transformed SE from both experiments were selected, and plants from transformed embryos of cv. “Canino” were recovered. In vitro, these plants displayed a high rooting capacity without the addition of auxins and showed the typical hairy root phenotype; a similar phenotype was observed in field trials. Additionally, transgenic plants showed a long juvenile phase and their development was altered, with morphological changes in the leaf blade, reduced leaf area and wider angles of insertion on the stem, reduced internode length and a higher number of internodes, and less apical dominance, showing a bushing structure [89]. Unfortunately, this trial had to be discontinued after intervention by the Italian Minister of Environment [90].

**Figure 4:**
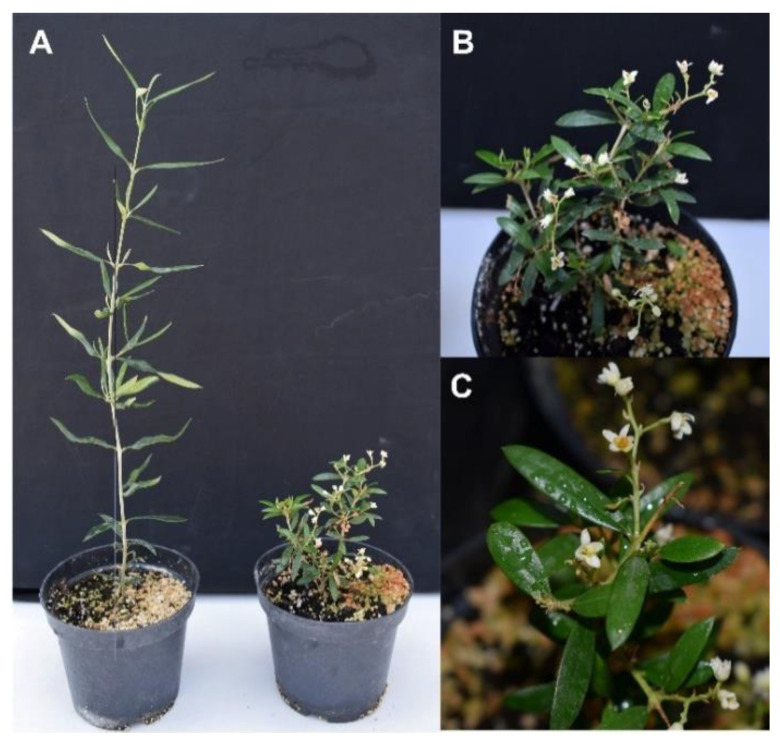
Early flowering olive plants expressing the *FT* gene *MtFTa1* from *Medicago truncatula*. (**A**) Control (left) and *MtFTa1*-transformed (right) olive plants. (**B**,**C**) Details of *MtFTa1*-transformed plants.

As in most woody species, the long juvenile period in olive, usually longer than 10 years, is one of the main bottlenecks in breeding programs. The overexpression of flowering genes, such as *Flowering Locus T* (*FT*), in transgenic plants shortens the juvenile period and induces continuous flowering. *FT* is expressed in the leaves under appropriate environmental conditions, and the protein is translocated via the phloem to the meristem, inducing flowering. *FT* overexpression has been attempted in a few woody species [91,92,93]. The use of these early flowering plants has been proposed as a novel method to reduce the generation time in woody species, accelerating breeding programs. In this system, called fast track breeding, an early flowering transgenic plant containing a single copy of the transgene is crossed with a parent line harboring the trait of interest. Offspring showing early flowering and the desired phenotype are selected and backcrossed with the elite commercial line. This process is repeated several times but, in the last backcross, early flowering plants are discarded, selecting the individuals with the desired trait but free of early flowering transgenes. Thus, improved plants devoid of transgenes could be generated in a short time. The proof-of-concept of this system was performed to obtain apple plants resistant to fire blight [94]. With the aim of accelerating the onset of flowering in olive, globular SE of a “Picual” embryogenic line were transformed with the *FT* gene from *Medicago truncatula*, *MtFTa1*, under the control of the *CaMV35S* promoter [69]. Some of the transgenic plants overexpressing *MtFTa1* showed the flowering of apical meristems in vitro. After acclimatization, plants of one of these transgenic lines flowered throughout the entire year in the glasshouse, with flowers being more abundant in spring (Figure 4). The growth pattern of the plants in some FT transgenic lines was altered, with a profuse axillary branching and reduced size [69]. Two of these lines showed the profuse branching phenotype but did not form flowers.

## 5. Future Prospects

Traditional olive breeding cannot fulfil current agricultural necessities. In this regard, genetic transformation and gene editing could be useful tools to generate novel genotypes with improved traits. The development of these biotechnological applications has been hampered by the recalcitrant nature of olive tissue. Protocols for gene manipulation in juvenile tissues are well established, although the recovery rates of transgenic plants are generally low and highly genotype dependent. The combined use of fluorescent markers with more efficient selection systems could help to increase transformation rates. The use of this methodology with SE of adult origin is still the main challenge for olive biotechnology, although successful results have been reported in one genotype. A more thorough understanding of the role played by osmotic [95,96] and demethylating [97,98] agents in improving embryogenic responses in recalcitrant genotypes could be of great help to overcome this problem. Moreover, the use of rejuvenation techniques has also been shown to increase the morphogenetic capacity of adult material; ca. in *Sequoia sempervirens*, the successive grafting of adult scions onto juvenile rootstocks allowed an improvement in the growth rate and rooting capacity of the adult scion [99]. These changes were linked to an increase in the levels of miR156, a key factor in the maintenance of juvenility in several woody perennials, olive included [100,101]. In any case and despite the limitations indicated above, some advances have been made in the genetic modification of important characteristics, such as disease tolerance, abiotic resistance, volatile production, and plant growth in juvenile and adult materials. The development and exploration of the olive genome sequence projects will surely speed up the discovery of novel genes with key roles in agronomical traits, opening new possibilities for olive improvement through cisgenesis, a genetic modification in which the genes used are from crossable species and which should be considered as a different breeding strategy than transgenesis [102]. In poplar, after the insertion of several copies of native genes related to gibberellin metabolism and signaling, trees with modified morphology and plant architecture were obtained [103]. Taking into account the importance of European countries in olive production and consumption and the strict rules regarding transgenic crop cultivation in the European Union (EU), the use of cisgenic material would become of particular importance for the acceptance of genetically modified olives; until then, olive transformation will basically remain as a useful tool for functional studies. Along this line, new outcomes in genomic information will also facilitate the application of gene editing technologies in this species.

## Figures and Tables

**Table 1:** Summary of olive transformation studies with genes of interest.

Trait	Gene	Olive Genotype	Type of Material Used	References
Fungal tolerance	*Osmotin* (*Nicotiana tabacum*)	Canino	Somatic embryos from adult origin	[64]
	*afp* (*Aspergillus giganteus*)	Picual	Somatic embryos from a mature seed	[65]
	*AtNPR1* (*Arabidopsis thaliana*)	Picual	Somatic embryos from a mature seed	[58]
Resistance to abiotic stress	*Osmotin* (*Nicotiana tabacum*)	Canino	Somatic embryos from adult origin	[64,66,67]
Olive oil quality	*13-HPL* (*O. europaea*)	Picual	Somatic embryos from a mature seed	[68]
Plant architecture/Flowering	*rolABC* (*A. rhizogenes*)	Canino	Somatic embryos from adult origin	[64]
	*MtFTa* (*Medicago truncatula*)	Picual	Somatic embryos from a mature seed	[69]

## Data Availability

Data sharing not applicable.
